# Crystal Orientation Effect on the Subsurface Deformation of Monocrystalline Germanium in Nanometric Cutting

**DOI:** 10.1186/s11671-017-2047-3

**Published:** 2017-04-26

**Authors:** Min Lai, Xiaodong Zhang, Fengzhou Fang

**Affiliations:** State Key Laboratory of Precision Measuring Technology and Instruments, Centre of MicroNano Manufacturing Technology, Tianjin University, Tianjin, 300072 China

**Keywords:** Monocrystalline germanium, Nanometric cutting, Amorphous, Anisotropy, Subsurface deformation, Molecular dynamics simulation

## Abstract

Molecular dynamics simulations of nanometric cutting on monocrystalline germanium are conducted to investigate the subsurface deformation during and after nanometric cutting. The continuous random network model of amorphous germanium is established by molecular dynamics simulation, and its characteristic parameters are extracted to compare with those of the machined deformed layer. The coordination number distribution and radial distribution function (RDF) show that the machined surface presents the similar amorphous state. The anisotropic subsurface deformation is studied by nanometric cutting on the (010), (101), and (111) crystal planes of germanium, respectively. The deformed structures are prone to extend along the 110 slip system, which leads to the difference in the shape and thickness of the deformed layer on various directions and crystal planes. On machined surface, the greater thickness of subsurface deformed layer induces the greater surface recovery height. In order to get the critical thickness limit of deformed layer on machined surface of germanium, the optimized cutting direction on each crystal plane is suggested according to the relevance of the nanometric cutting to the nanoindentation.

## Background

In recent years, the accuracy and the dimension of ultra-precision machining have reached nanoscale along with the development of science and technology. A grasp of deformation mechanism in the material during nanometric processing becomes essential to achieve higher surface finishing and damage-free subsurface. Monocrystalline germanium, a group IV elemental semiconductor, has been widely used in the fields of solar cell, infrared optics, and so on. As it has the periodic ordered arrangement of diamond structure, which is similar to silicon, the anisotropy feature of germanium during nano-machining should be paid more attention to. In fact, the mechanism of subsurface deformation and material removal of single crystal is strongly influenced by the crystallographic orientation.

A number of studies have been carried out to investigate the crystal orientation effects during the single-crystal machining. Komanduri et al. [1, 2] studied the effect of crystal orientation on the nature of deformation by molecular dynamics (MD) simulation in copper and aluminum cutting and proposed different models of plastic deformation in shear zone. Some other researchers found that the average roughness and surface damage of single-crystal copper and silicon varied with crystal orientation in experiments [3, 4]. Hung et al. [3] discovered that damages of silicon would originate/terminate at one of the {111}, 110 slip systems. Blacklet et al. [5] applied a line-force stress model to predict the variation of damage on the different crystal faces of germanium. According to the previous researches, monocrystalline silicon underwent phase transformation from diamond cubic structure to Si-II (β-tin-Si) or amorphous structure in the loading process of nanometric cutting and nanoindentation [6–12]. In the unloading period, the β-tin-Si transformed to Si-XII/Si-III or amorphous silicon according to the process parameters. The existence of Si-XII, Si-III, and amorphous silicon in the machined region were confirmed by a great deal of results from molecular dynamics simulations and experimental study [7, 8, 10–14]. In the case of germanium, attentions were focused on the experimental measurements of material response during nanoindentation. The methods include electrical resistance test [15], scanning electron microscopy [16], cross-sectional transmission electron microscopy [16–19], Raman spectroscopy [16–21], and X-ray diffraction [19]. There is some controversy as to whether shear-induced plasticity or high-pressure phase transformation is the dominant deformation of monocrystalline germanium in nanoindentation. Recently, MD simulation has been used to study the nanoindentation of germanium film, and the pressure-induced phase transformation was found to be the dominant deformation mechanism of monocrystalline germanium instead of dislocation-assisted plasticity [22]. Our previous MD simulation about the machined surface of germanium after nanometric cutting and nanoindentation showed that the deformed layer after machining presented amorphous structure [23, 24]. So far, the researches about the mechanism of subsurface deformation in germanium during nanometric cutting have rarely been found, as well as about the difference of subsurface deformation induced by anisotropic of monocrystalline germanium. In fact, the investigations about the anisotropic behavior of single-crystal brittle materials in nanometric cutting have focused on the effects of crystal orientation on the limit of ductile machining (initial crack) instead on the subsurface deformation layer of phase transformation at present. Subsurface damages, including the structural deformation, residual stress, and cracks, have a great potential effect on the performance and service life of high-precision optics. The study on the deformation mechanism of germanium in nanometric cutting can provide theoretical basis for developing the damage-less nanometric machining method for germanium optics.

In the present study, molecular dynamic simulations of nanometric cutting on germanium are conducted to investigate the subsurface deformation during and after machining. The cutting directions include several combinations of orientation and plane, and the structures in detail at the atomic level are disclosed and computed accordingly. The relationship between the structure of deformed subsurface and crystal orientation is observed and analyzed. In order to get the thinnest subsurface deformed layer, the machined directions on specific planes of germanium are suggested at the end of this study.

## Methods

**Fig. 1:**
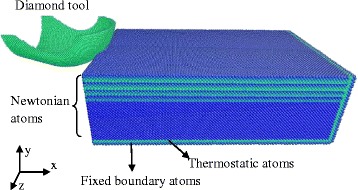
MD simulation model of nanometric cutting

The three-dimensional MD simulation model consists of the germanium substrate and a diamond tool, as shown in Fig. 1 The workpiece has a size of 45 nm × 27 nm × 12 nm. The atoms in the bottom and retracting side layers keep fixed to restrain the motion range of other atoms in the workpiece, avoiding the translation induced by cutting force, which disagrees with the real cutting condition and is strongly undesirable in MD simulation. The layers neighboring the fixed atoms are called thermostat atoms, and their computing temperature is kept at 293 K. The rest of the atoms belong to the Newtonian region. In the circumstance of predefined potential field, the motion of the atoms in this area obeys the classical Newton’s second law. The three-dimensional diamond tool has an edge radius of 10 nm and tool nose radius of 10 nm, which is set up as the shape of a real cutter [25]. To simplify the simulation process, the diamond tool with 64,769 atoms is regarded as a rigid body. Since germanium is a covalent crystal, which is the same with silicon, the Tersoff potential is adopted to depict the interaction among the inner atoms [22–24, 26]. With regard to the interaction potential between diamond tool and workpiece, the Morse potential is used because the Morse potential is simple and computationally inexpensive and it has been used for several similar studies previously [23].

**Fig. 2:**
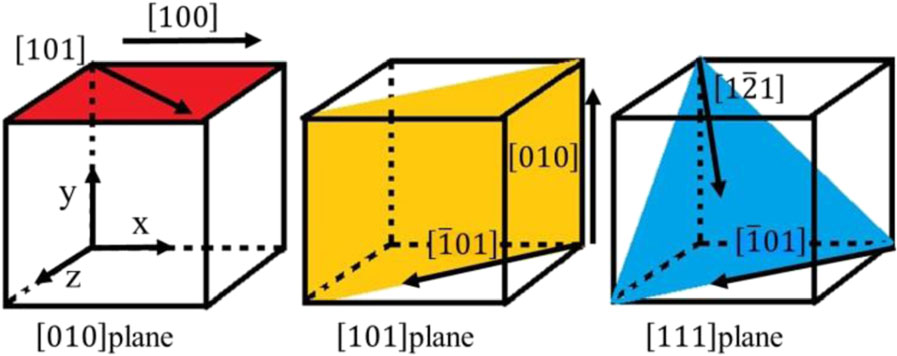
Schematic diagram of cutting directions on the crystal planes of monocrystalline germanium

To investigate the effect of crystal orientation on the subsurface deformation in nanometric cutting, three typical crystallographic planes including the (010), (101), and (111) faces are designed as the machined surface and the cutting directions are shown in Fig. 2. The main simulation parameters and other model conditions are listed in the Table 1.

**Table 1:** Model condition and simulation parameters

Workpiece dimensions	45 nm × 27 nm × 12 nm
Tool edge radius	10 nm
Tool nose radius	10 nm
Tool clearance angle	15°
Cutting direction	[100] and [101] on the (010) plane
	$\left[\overline{1}01\right]$ and [010] on the (101) plane
	$\left[1\overline{2}1\right]$ and $\left[\overline{1}01\right]$ on the (111) plane
Depth of cut	3 nm
Initial bulk temperature	293 K
Parameters of C-Ge morse potential	
De = 0.125778 eV, *α* = 2.58219 Å^−1^, *r*_0_ = 2.2324 Å [23]	

## Results

### Method to Estimate the Amorphous Germanium

Many researchers have found that monocrystalline silicon underwent amorphization in nanometric cutting and nanoindentation by MD simulation and experimental study [7, 8, 10–14]. Monocrystalline germanium has similar phase transformations with silicon under pressure, and it was found that monocrystalline germanium become amorphous state in the machined region in nanometric cutting and nanoindentation by MD simulation [22–24]. Usually, the criterion to estimate the amorphization of single crystal in MD simulation in nanometric machining is to observe the disordered degree of atomic structure directly [7, 8, 13]. Then, the distribution of atomic bond length in addition to the observed atomic structure was proposed to determine the existence of amorphization in the machined surface of germanium in previous study [23]. In order to characterize the amorphous germanium scrupulously, the MD simulation of amorphous germanium is conducted and its characteristic parameters are extracted to compare with those of the nanometric machined surface in this study. The continuous random network (CRN) model is a widely accepted description of the atomic arrangement in amorphous tetrahedrally coordinated semiconductors. This model has a high degree of short-range order and no long-range order. Especially, the short-range neighbor distances of the model are in excellent agreement with the results from the experimental test of extended X-ray absorption fine structure [27].

The approach to structure the CRN model of amorphous germanium is as follows. A monocrystalline germanium substrate is modeled with the size of 30*a* × 30*a* × 30*a* (*a* = 5.657 Å) and with a periodic boundary condition. After relaxation at initial 293 K, germanium is heated to 4500 K slowly, which is much higher than the melting temperature of monocrystalline germanium in MD simulation so that the germanium presents liquid state. After that, the workpiece is cooled to room temperature (293 K) quickly for imitating the quenching and then relax the model for a while. Thus, the stable amorphous germanium is modeled. As the time span is limited in simulations, the cooling rate can be high enough to get amorphous germanium. In addition, the temperature mentioned above is calculated in the simulation and cannot be compared with the real condition directly. The melting temperature of germanium in MD simulation with Tersoff potential is about 3300 K [27], which is much higher than the real melting temperature of 936 K. As a result, 4500 K for the top heated temperature is merely to make sure that germanium becomes a complete molten state and then the amorphous germanium can be obtained through quenching.

**Fig. 3:**
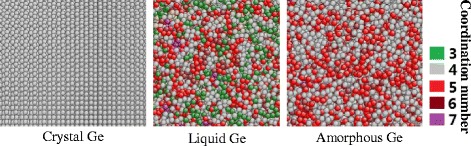
Coordination number distributions of monocrystalline, liquid, and amorphous germanium

Figure 3 shows the snapshots and the coordination number distributions of monocrystalline, liquid, and amorphous germanium. The liquid germanium has the widest range of coordination number from three to eight, while the amorphous germanium consists of fourfold coordinated atoms, fivefold coordinated atoms, and a small amount of sixfold coordinated atoms.

**Fig. 4:**
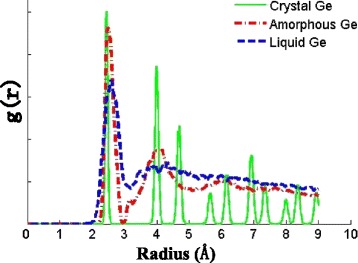
Radial distribution function (RDF) of the monocrystalline, liquid, and amorphous germanium

Figure 4 displays the radial distribution function (RDF) of the monocrystalline, liquid, and amorphous germanium. The structure of monocrystalline germanium is in order periodically while the amorphous germanium shows a strong short-range order and virtually no medium and long-range order. It can be found that the nearest neighbor distance of the amorphous germanium has a slight displacement relative to the monocrystalline germanium, and the peak value is about 2.48 Å [28], which is larger than the value of monocrystalline germanium, i.e., 2.45 Å. The peak curve of the second neighbor distance also appears displacement and extension. As for the further distance, amorphous germanium presents disordered structure, which is similar to liquid germanium.

### Phase Transformation after Nanometric Cutting

**Fig. 5:**
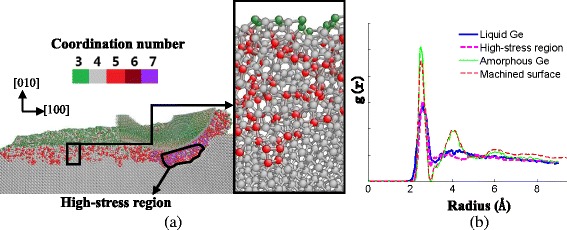
**a** Atomic structure and coordination number distribution of machined surface of germanium, **b** RDF

Figure 5a shows the atomic structure and coordination number distribution of machined surface with cutting direction of [100] on the (010) plane of germanium. It can be seen that the coordination numbers of the deformed layer consist of plentiful four, less five, and rare six except for the surface atoms, which agree with the amorphous germanium obtained above. For comparison, the RDF of the high-stress region during the nanometric cutting of germanium, liquid germanium, machined surface, and CNR model of amorphous germanium are plotted together, as shown in Fig. 5b. The peak location and peak width of machined surface almost coincide with those of amorphous germanium in short range. Meanwhile, both of them present no long-range order. In consideration of the two points above, the conclusion that the deformed layers of germanium present amorphous structure after nanometric cutting is drawn in this study. During the nanometric cutting of germanium, the high compressive stress produced by the effective negative rake face enables the crystal structure of the regions forward beneath the tool to change into the complete amorphous structure. The RDF of this region is similar to that of liquid germanium. In fact, the previous MD simulation investigation using the Tersoff potential indicated that a gradual low-density amorphous to high-density amorphous transformation occurred under pressure and the high-density amorphous phase was similar to the liquid germanium [29]. Thus, monocrystalline germanium mainly undergoes direct amorphization in nanometric cutting besides the phase transformation from diamond cubic structure to β-tin phase [23, 24]. In addition, the germanium material under and in front of the tool presents high-density amorphous structure, which is similar to the liquid germanium. This part of the material undergoes plastic-like flow as the tool continues moving and extrudes out from the tool rake face like the fluid, forming the chips. The process mentioned above presents a good agreement with Fang’s model of the extrusion mechanism in nanometric cutting [14, 26, 30].

### Surface and Subsurface Deformation on Different Crystallographic Planes

#### (010) Crystal Face

**Fig. 6:**
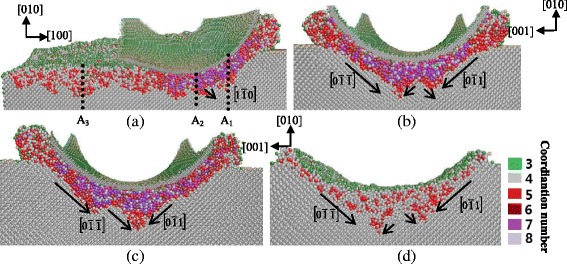
Cross-sectional views of the subsurface deformation when nano-cutting along the [100] direction on the (010) plane. **a** On the (001) plane, passing along the [100] direction through the middle of the tool. **b** On the (100) plane, passing through *A*_*1*_. **c** On the (100) plane, passing through *A*_*2*_. **d** On the (100) plane, passing through *A*_*3*_

In the case of cutting along the [100] direction on the (010) surface, the serious deformation is observed in the region beneath the tool, which is proved to be amorphous structure. Previous study showed that a large area of phase transformation from diamond cubic structure to β-tin-Ge was obviously observed in the subsurface region of nanoindentation on the (100) plane by MD simulation [24]. However, most of the pressure region underneath is observed to be amorphous state on the same crystal plane in nanometric cutting besides the limited phase transformation mentioned above, as displayed in Fig. 6. The previous study also showed that the phase transformation from diamond cubic structure to β-tin-Ge was mostly found in nanoindentation on the (100) plane while only the direct amorphization was observed in nanoindentation on the (110) and (111) planes [24], which means that this kind of phase transformation of germanium in nano-machining happens only in specific directions of uniaxial pressure except the hydrostatic compression [31].

**Fig. 7:**
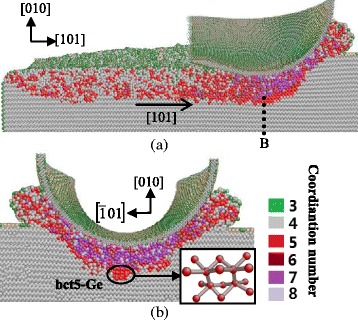
Cross-sectional views of the subsurface deformation when nano-cutting along the [101] direction on the (010) plane. **a** On the $\left(\overline{1}01\right)$ plane, passing along the [101] direction through the middle of the tool. **b** On the (101) plane, passing through *B*

When the cutting direction is along the [101] direction on the (010) plane, most of the germanium atoms neighboring beneath the tool present amorphous state. Unlike the subsurface deformation with the cutting direction of [010], the bct5-Ge structure is observed under this high-pressure region, as shown in Fig. 7. The pressure direction and stress strength of the cut area atoms change along with the movement of the tool in nanometric cutting, in contrast to the simplex pressure direction of subsurface beneath the indenter in nanoindentation. Especially, the change rate increases with the decrease of distance between the workpiece and tool. As a result, the subsurface deformation of germanium in nanometric cutting becomes complicated and the materials under this pressure turn into the amorphous structure directly because of the complex pressure.

According to the preceding analysis, the machined region forms an amorphous layer with a certain depth. The transformed region extends mainly along the $\left[0\overline{1}\overline{1}\right]$ or $\left[0\overline{1}1\right]$ direction, as shown in Fig. 6b–d. The cross-sectional view on the (001) plane (Fig. 6a) also shows that the boundary between transformed phase and diamond cubic structure is along the $\left[1\overline{1}0\right]$ direction. This phenomenon is in accordance with those of silicon and germanium in nanoindentation by MD simulation [8, 24]. All of the directions belong to the same germanium’s slip direction of 110. The subsurface deformed layer displayed in Fig. 7 shows the same tendency.

#### (101) Crystal Face

**Fig. 8:**
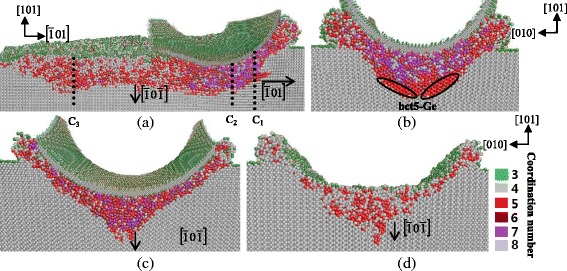
Cross-sectional views of the subsurface deformation when nano-cutting along the $\left[\overline{1}01\right]$ direction on the (101) plane. **a** On the (010) plane, passing through the middle of the tool. **b** On the $\left(\overline{1}01\right)$ plane, passing through *C*_*1*_. **c** On the $\left(\overline{1}01\right)$ plane, passing through *C*_*2*_. **d** On the $\left(\overline{1}01\right)$ plane, passing through *C*_*3*_

Figure 8a–d is cross-sectional views on different planes and positions when cutting along the $\left[\overline{1}01\right]$ direction on the (101) plane. The machined surface presents amorphous phase, which is the same with that along the other cutting directions in this study. The deformed structures tend to extend along the $\left[\overline{1}01\right]$ and $\left[\overline{1}0\overline{1}\right]$ orientations. Since the $\left[\overline{1}0\overline{1}\right]$ direction is normal to the machined surface and the stress distribution in the region contacting the tool is nonuniform, the thickness of the deformed layer on the $\left(\overline{1}01\right)$ plane differs in various positions. In the subsurface area beneath and in front of the tool, the bct5-Ge with a coordination number of five is observed.

**Fig. 9:**
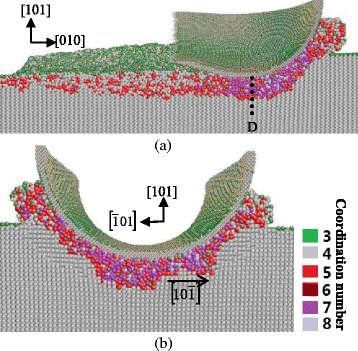
Cross-sectional views of the subsurface deformation when nano-cutting along the [010] direction on the (101) plane. **a** On the $\left(\overline{1}01\right)$ plane, passing through the middle of the tool. **b** On the (010) plane, passing through *D*

Figure 9 shows the cross-sectional snapshots of subsurface deformation with cutting direction of [010] on the (101) plane. Since the extending direction of deformed layer is $\left[10\overline{1}\right]$, which is parallel to the machined surface, the subsurface deformation in this cutting condition becomes more uniform. Although cutting on the same crystal plane, the directions of main cutting forces are not the same with different cutting directions. As a result, the position relation of cutting and subsurface-deformation-extending direction change, which induces the difference in the subsurface deformation.

#### (111) Crystal Face

**Fig. 10:**
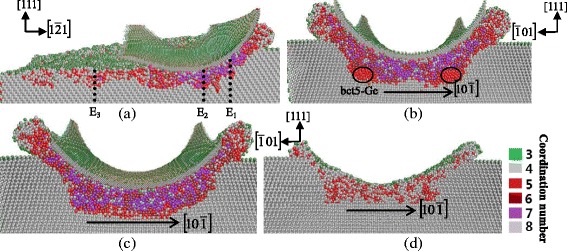
Cross-sectional views of the subsurface deformation when nano-cutting along the $\left[1\overline{2}1\right]$ direction on the (111) plane. **a** On the $\left[\overline{1}01\right]$ germanium surface, passing through the middle of the tool. **b** On the $\left(1\overline{2}1\right)$ plane, passing through *E*_*1*_. **c** On the $\left(1\overline{2}1\right)$ plane, passing through *E*_*2*_. **d** On the $\left(1\overline{2}1\right)$ plane, passing through *E*_*3*_

**Fig. 11:**
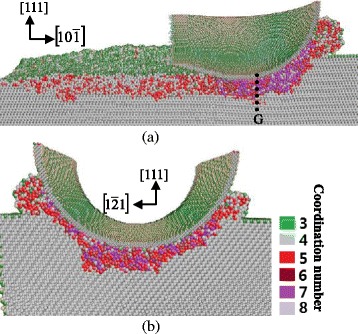
Cross-sectional views of the subsurface deformation when nano-cutting along the $\left[10\overline{1}\right]$ direction on the (111) plane. **a** On the $\left[1\overline{2}1\right]$ plane, passing through the middle of the tool. **b** On the $\left(10\overline{1}\right)$ plane, passing through *G*

Figures 10 and 11 are the cross-sectional views of cutting along the $\left[1\overline{2}1\right]$ and $\left[10\overline{1}\right]$ direction, respectively, on the (111) plane. When the cutting direction is $\left[1\overline{2}1\right]$, the tangent cut area under the tool performs amorphous structure and transformed phase of bct5-Ge appears at both sides under this region. Figure 10b–d is cross-sectional views on the $\left(1\overline{2}1\right)$ plane. These figures show that the boundary of deformed region and diamond cubic structure is almost parallel to the $\left[10\overline{1}\right]$ direction, which belongs to the 110 slip system. Therefore, the machined surface has a relative uniform depth of amorphous structure from the view on the $\left(1\overline{2}1\right)$ plane. Similarly, the subsurface deformation of cutting along the $\left[10\overline{1}\right]$ direction also shows a relative uniform in depth, as displayed in Fig. 11. These results can be explained by the fact that the (111) surface is the slip plane of germanium and has the largest interplanar crystal spacing. Therefore, the germanium atoms tend to move laterally instead of shifting normally with loading on the slip plane. Consequently, the deformed structures are apt to extend along the (111) surface, causing the relative uniform subsurface deformation.

## Discussion

The thickness of deformed layer and the surface recovery height when cutting on various lattice planes are measured in this study. Since the machined surface is not flat because the form of three-dimensional tool would print as the cut mark, the thickness of deformed layer is defined as the distance from the lowest position of the surface, namely, the middle of the cut mark, to the deepest location of the deformed layer in workpiece. Assume the bottom of the tool contact closely to the materials, the surface recovery height is defined as the distance from the bottom of the tool during cutting to the middle position of cut groove after machining. In order to obtain the details of subsurface deformation when cutting along different orientations, the thickness of deformed layer and surface recovery height of machined surface are measured from 17 equally spaced cross sections of machined surface, which are perpendicular to the cutting direction.

**Fig. 12:**
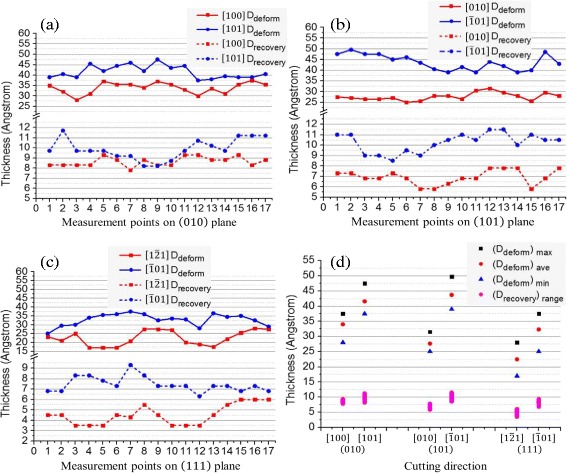
The thickness of deformed layer (*D*_*deform*_) and recovery height (*D*_*recovery*_) with different cutting orientations. **a** Cutting alone the [100] and [101] direction on the (010) plane. **b** Cutting alone the [010] and $\left[\overline{1}01\right]$ direction on the (101) plane. **c** Cutting alone the $\left[1\overline{2}1\right]$ and $\left[\overline{1}01\right]$ direction on the (111) plane. **d** The maximum, average, and minimum values of *D*_*deform*_ and the range of *D*_*recovery*_

Figure 12 shows the measurement results. We can see that with the same cutting depth of 3 nm, the thickness of deformed layer on machined surface are quite different when cutting along different crystal orientations on the same crystal plane. On the (010) plane, the average thickness of deformed layer with cutting direction of [101] is about 1 nm thicker than that with cutting along the [010] direction. On the (101) plane, the average thickness of deformed layer when cutting along the $\left[\overline{1}01\right]$ direction is about 1.5 nm thicker than that with cutting along the [010] direction. On the (111) plane, the average thickness of deformed layer when cutting along the $\left[10\overline{1}\right]$ direction is 1 nm thicker than that with cutting along the $\left[1\overline{2}1\right]$ direction. In addition, it can be found that the recovery height increases with increment of the thickness of deformed layer on machined surface. Except for the elastic recovery, the plastic recovery is mainly induced by the difference in density of the two structural phases during and after cutting on the machined surface [24]. During nanometric cutting, the subsurface area underneath the tool presents the high-density amorphous structures, as shown in Fig. 5. After machining, the sub/surface presents the normal amorphous germanium, which has the lower density. Thus, this phase transformation leads to the change of volume, inducing the surface recovery. Moreover, the normal amorphous germanium has the 20% less density than the monocrystalline germanium, which means the phase transformation from monocrystalline to the amorphous structure of germanium will lead to the surface recovery. As a result, the thicker deformed layer leads to the greater volume difference in phase-transformed material before and after unloading, inducing a bigger recovery on the machined surface of germanium.

Generally, the controllable surface recovery is highly desirable in the ultra-precision machining in order to ensure that the machined surface is as close to the designed forming as possible. In nanometric cutting, processing in the nanometer range and extremely high surface accuracy are needed. The uncontrollable surface recovery may be fatal to the surface integrity, which would be counterproductive to the goal of achieving controllable nanometric cutting. Therefore, less deformed layer on machined surface is required so as to get the high surface accuracy when cutting on monocrystalline germanium in nanoscale directly.

**Fig. 13:**
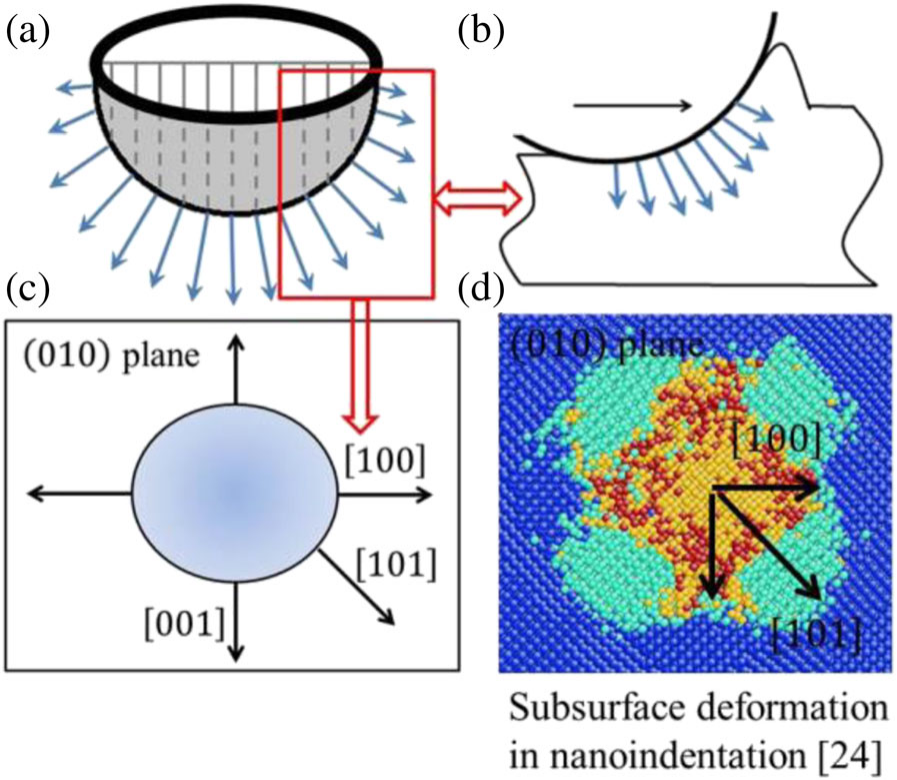
**a** The stress directions on the normal cross section in nanoindentation with a spherical indenter. **b** The stress directions on the normal cross section in nanometric cutting with a tool nose radius. **c** Schematic illustration of orientations on the (010) crystal plane of monocrystalline germanium. **d** The subsurface deformation induced by nanoindentation on the (010) plane of germanium

Usually, nanoindentation is used to study the mechanical properties of materials in nanometer scale, such as nano-hardness and phase transformation under stress [8, 22, 24]. Figure 13 shows the stress directions on the cross section in nanoindentation with a spherical indenter. In nanometric cutting, the cross section of contact region, which is parallel to the cutting direction, is considered to be the form of circular arc because the tool edge radius is much larger than the depth of cut [14, 26, 32]. When the tool moves along a certain direction, the subsurface stress distribution ought to be similar to that of corresponding direction in nanoindentation on the same crystal plane with a spherical indenter. Previous study indicated that the transformed phase distributions in subsurface are different with nanoindentation on various crystal planes for silicon and germanium. Even on the same crystal plane, the phase transformations are not the same along different orientations [8, 24]. For example, when cutting along the [100] direction on the (010) plane of germanium, the stress distribution beneath the tool should be similar to that around the [100] orientation in nanoindentation on the same crystal plane, as shown in Fig. 13. In nanoindentation on the (010) plane of germanium, the subsurface materials around the 101 direction undergo the direct amorphization and the phase transformation from diamond cubic structure to β-tin phase, and the extensive phase transformation from diamond cubic structure to bct5-Ge occurs [24]. The bct5-Ge structure is believed to be the transition state between the diamond cubic structure and β-tin phase and related to amorphous structure [33]. The stress intensity of each direction is the same with a spherical indenter in nanoindentation. Therefore, the simulation results of nanoindentation on the (010) plane of germanium show that the range of bct5-Ge structure extending along the 101 direction is much more extensive than the range of amorphous germanium extending along the 100 direction with the same stress intensity [24], which means much more subsurface deformation of germanium appears with the stress along the 101 direction instead of along other directions on the (010) crystal plane. Accordingly, the thickness of deformed layer with cutting along the [101] direction is thicker than that with cutting along the [010] direction in nanometric cutting on the (010) crystal plane of germanium. Analogously, on the (101) crystal plane, the range of bct5-Ge structure extending along the $\overline{1}01$ direction is much larger than the range of amorphous germanium extending along the 010 direction in nanoindentation. On the same crystal plane, the average thickness of deformed layer with cutting along the $\left[\overline{1}01\right]$ direction is much greater than that with cutting along the [010] direction. The similar conclusions also hold true for machining on the (111) crystal plane of germanium.

Generally, the thicknesses of subsurface deformation are different from each other with various combinations of cutting crystal plane and orientation in nanometric cutting of monocrystalline germanium because of its anisotropy. Thus, a large number of simulations or experiments may be needed for investigating the relative depth of subsurface deformation with cutting directions, which is time-consuming. According to the analysis above, the relative depth of subsurface deformation when nano-cutting along different directions on the monocrystalline germanium can be obtained from the transformed phase distribution of the subsurface with nanoindentation on the same crystal plane with a spherical indenter, instead of extremely large numbers of simulations or experiments on nanometric cutting. That is, the larger extending range of transformed phase on the subsurface around a certain direction in nanoindentation, the greater thickness of deformed layer would occur with nano-cutting along the same direction on the same crystal plane of germanium. Thus, it helps to select the appropriate cutting direction considering the demands for the thickness of deformed layer in nanometric cutting of monocrystalline germanium. For example, in order to get the machined surface with the thinnest thickness of deformed layer in nanometric cutting, the cutting directions of 100 on the (010) crystal plane, 010 on the (101) crystal plane, and $\left[1\overline{2}1\right]\left[11\overline{2}\right]\left[\overline{2}11\right]$ on the (111) crystal plane of germanium are suggested.

## Conclusions

The MD simulations of nanometric cutting on the (010), (101), and (111) planes of monocrystalline germanium are carried out in this study. The CRN model of amorphous germanium is established to contrastively analyze the structure of germanium on the machined surface. The anisotropic behaviors in subsurface deformation are investigated and the conclusions are as follows:

1. Compared with the CRN model of amorphous germanium, the RDF and coordination number distribution show that the machined surface of germanium presents the similar amorphous state.
2. The subsurface deformed structures of germanium after nanometric cutting tend to extend along the 110 slip system. The surface recovery height increases with the increment of thickness of subsurface deformed layer on machined surface.
3. The thickness of subsurface deformed layer on the machined surface varies with different cutting orientations. According to the analyzed relevance of subsurface deformation in nanometric cutting to nanoindentation, nanometric cutting along the 100 direction on the (010) crystal plane, 010 direction on the (101) crystal plane, and $\left[1\overline{2}1\right]\left[11\overline{2}\right]\left[\overline{2}11\right]$ directions on the (111) crystal plane on germanium can lead to the thinnest thickness of deformed layer on the machined surface.

## Abbreviations

CRN: Continuous random network
MD: Molecular dynamics
RDF: Radial distribution function
